# Development of an Extended Cardiovascular SOFA Score Component Reflecting Cardiac Dysfunction with Improved Survival Prediction in Sepsis: An Exploratory Analysis in the Sepsis and Elevated Troponin (SET) Study

**DOI:** 10.1177/08850666241282294

**Published:** 2024-10-01

**Authors:** S. Lörstad, Y. Wang, S. Tehrani, S. Shekarestan, P. Åstrand, P. Gille-Johnson, T. Jernberg, J. Persson

**Affiliations:** 1 Division of Internal Medicine, Department of Clinical Sciences, Karolinska Institutet, 72227 Danderyd University Hospital, Stockholm, Sweden; 2 Department of Clinical Sciences, 72227 Danderyd University Hospital, Karolinska Institutet, Stockholm, Sweden; 3 Division of Cardiovascular Medicine, Department of Clinical Sciences, Karolinska Institutet, 72227 Danderyd University Hospital, Stockholm, Sweden; 4 Internal Medicine Clinic, 72227 Danderyd University Hospital, Stockholm, Sweden; 5 Infectious Diseases Clinic, 72227 Danderyd University Hospital, Stockholm, Sweden

**Keywords:** SOFA, sepsis, troponin, NT-proBNP, atrial fibrillation, myocardial injury

## Abstract

**Introduction:**

The cardiovascular component of the Sequential Organ Failure Assessment (SOFA) score does not correspond with contemporary clinical practice in sepsis or identify impaired cardiac function. Our aim was to develop a modified cardiovascular SOFA component that reflects cardiac dysfunction and improves the SOFA score's 30-day mortality discrimination.

**Methods:**

A cohort of sepsis patients from a previous study was divided into a training (n = 250) and test cohort (n = 253). Nine widely available measures of cardiovascular function were screened for association with 30-day mortality using natural cubic spline. High-sensitivity cardiac troponin T (hs-cTnT), N-terminal pro B-type natriuretic peptide (NT-proBNP) and heart rate (HR) were transformed into ordinal variables (0-4 points). The presence of atrial fibrillation (AF) was assigned two points. The SOFA score was extended by adding the variable points in different weights and combinations. The best-performing cardiac-extended model (CE-SOFA) was evaluated in the test cohort. Improved prognostic discrimination and calibration were assessed using logistic regression, area under receiver operating characteristic curves (AUC), Net Reclassification Improvement (NRI) index, and DeLong and Hoshmer-Lemeshow tests.

**Results:**

In the training cohort, all differently weighted and combined models using hs-cTnT, NT-proBNP and AF points added to the SOFA score showed improved discriminative ability (AUC 0.67-0.75) compared to the SOFA score (AUC 0.62; NRI *P *< .001; DeLong *P* ≤ .001). In the test cohort, CE-SOFA demonstrated improved 30-day mortality discrimination compared to the SOFA score (AUC 0.72 vs 0.68), exhibiting good calibration and significantly improved discrimination using the NRI index (*P *= .009) but not the DeLong test (*P *= .142).

**Conclusions:**

The CE-SOFA model reflects cardiac dysfunction and improves 30-day mortality discrimination in sepsis. External validation is the next step to further substantiate a revised cardiovascular component in a future SOFA 2.0.

## Introduction

The Sequential Organ Failure Assessment (SOFA) score evaluates the severity of organ dysfunction (0-4 points) across six different organ systems. Although originally developed for the clinical evaluation of multiorgan dysfunction in sepsis patients, the SOFA score has since been applied to quantify disease severity in a wide range of critical conditions.^1–3^ The *Sepsis-3* task force reinforced the sepsis connection by integrating the SOFA score to evaluate disease severity into the latest definitions of sepsis and septic shock.^4^

The SOFA score was conceived to describe and quantify organ dysfunction or failure over time rather than predict mortality.^3^ However, it is well-established that mortality rates correlate directly with the severity of individual organ dysfunction as well as the number of organ system failures.^2,3,5,6^ As a result, the prognostic performance of the SOFA score has been extensively assessed and consistently demonstrates comparable predictive performance to that of other well-established prognostic scores.^1,7,8^ More recently, research has turned towards examining the predictive ability of each separate SOFA component, revealing that the sub-scores from the six different organ systems possess different prognostic abilities and weights.^2,6,9,10^

In recent years, there has been growing acknowledgement of the need to revise the SOFA score to align it with current clinical practices and biomarker developments.^9,11–13^ The cardiovascular component, for example, no longer reflects contemporary recommendations for vasoactive agent administration in septic or cardiogenic shock.^11,12,14^ Furthermore, studies have indicated that measurements of mean arterial pressure (MAP) alone do not adequately reflect cardiovascular dysfunction.^11,12,15–17^

The clinical evaluation of organ dysfunction and failure based on simple, continuous, widely available, and organ-specific variables that can be followed over time was central to the original SOFA conception.^3,9,12^ While the creators of the SOFA score emphasized that the criteria for each component should not be considered definitive, they highlighted the importance of retaining the fundamental principles of widely available and organ-specific variables in future revisions.^3,11^ The importance of retaining these fundamental characteristics to ensure easy and economically feasible clinical implementation has been emphasised in the move towards an updated SOFA 2.0.^11^

This is an exploratory study in which we have screened and evaluated widely available and simple measures and biomarkers of cardiovascular function using a cohort of critically ill sepsis patients. We hypothesized that the addition of stratified points for one or more cardiac-specific variables would improve the current cardiovascular SOFA component's ability to reflect concurrent myocardial injury and dysfunction and enhance the composite SOFA score's 30-day mortality discrimination in sepsis.

## Methods

### Derivation Cohort

The design, inclusion and exclusion criteria for the Sepsis and Elevated Troponin (SET) study have been previously described in detail.^18^ Approval for the study protocol was obtained from the Swedish Ethical Review Authority (2020-00137, 2021-03468). In summary, the cohort consisted of 586 adult patients treated for sepsis at the Danderyd University Hospital, Stockholm, Sweden between March 2012, and September 2021. A flow chart describing the patient inclusion process for the SET study is shown in Supplementary Figure S1. Patients meeting the inclusion criteria were identified retrospectively, and all: (i) required vasopressor support, (ii) fulfilled the *Sepsis-3* criteria for sepsis or septic shock^4^ and (iii) had at least one sampled high-sensitivity cardiac troponin T (hs-cTnT) value following sepsis onset. All data were extracted from hospital electronic health records (EHR).^18^ Patients lacking sampled N-terminal pro B-type natriuretic peptide (NT-proBNP) value (n = 83) were then excluded from the current study, resulting in a final study cohort of 503 patients.

### Training and Test Cohorts

The primary endpoint was improved all-cause 30-day mortality discrimination by the cardiac-extended SOFA models compared with the reference SOFA score. Previous literature indicated that the reference SOFA score would yield an anticipated area under the receiver operator characteristic curve (AUC) score of 0.70 for 30-day mortality.^7,19,20^ Based on this, the sample size calculation determined that 227 subjects were required to achieve an AUC of 0.74, with a significance level of 5% and a power of 80%.

The derivation cohort was randomly divided into a training cohort (n = 250) and test cohort (n = 253) using Microsoft 365 Excel^®^. All experimental analyses were performed in the training cohort. The final model was analysed in the test cohort once all results using the training cohort were completed. Missing data in both cohorts were negligible.

### Clinical Definitions

Sepsis onset was defined as the documented time in the EHR when the patient's vital signs became abnormal. The reference SOFA score was the total SOFA score (the sum of all six SOFA component sub-scores) calculated upon admission to either the Intermediate Care Unit (IMCU) or the Intensive Care Unit (ICU) following sepsis onset. Severity of illness was assessed using the SOFA score, Acute Physiology and Chronic Health Evaluation II score (APACHE II)^21^ and Kidney Disease: Improving Global Outcomes (KDIGO) staging criteria.^22^

### Cardiac Biomarkers

Plasma hs-cTnT (Roche Diagnostics, Elecsys Assay^23^) and plasma NT-proBNP (Roche Diagnostics, Elecsys Assay^24^) were analysed at the Karolinska University Laboratory, Stockholm using electrochemiluminescence technology. Hs-cTnT ≥15 ng/L was defined as elevated.^25^ The absence of acute heart failure was defined as NT-proBNP <300 ng.^26^ All laboratory values included in the analysis were collected within two hours before admission to the IMCU/ICU and up to 24 h after admission. The first hs-cTnT and NT-proBNP values obtained within this time frame were selected and used in all analyses.

### Statistical Analysis

Demographics and other baseline characteristics are presented as frequencies and percentages for categorical variables and the mean ± standard deviation (SD) for continuous variables. Non-normally distributed continuous and categorical variables are summarised as the median value and interquartile range (IQR). Statistical significance for the continuous variables was assessed using one-way ANOVA for normally distributed data and the Mann-Whitney *U* test for non-normally distributed data. The Pearson Chi-Square test was employed for categorical variables.

#### Step 1: Cardiovascular Variable Selection and Transformation

We used natural cubic spline transformation to model the effect of nine continuous predictors of cardiovascular function on 30-day mortality: hs-cTnT; NT-proBNP; heart rate (HR); systolic blood pressure (SBP); diastolic blood pressure (DBP); MAP (DBP + 1/3[SBP-DBP]); pulse pressure (PP; SBP-DBP); rate pressure product (RPP; HR × SBP) and proportional pulse pressure (PPP; PP/SBP). The number of knots for the natural cubic splines was determined by selecting a degree of freedom, which in turn split the data into quantiles. The choice of degree of freedom was based on minimizing the Akaike Information Criterion (AIC). Due to their right-skewed distributions, both hs-cTnT and NT-proBNP were logarithmically transformed. The HR, SBP and DBP values registered in the EHR upon admission to the IMCU/ICU were analysed. The discriminative ability of each continuous variable was evaluated using the AUC. The continuous variables demonstrating an association with 30-day mortality were transformed into ordinal variables (point groups) using logistic regression models. These variables were categorized on a scale of 0 to 4 points, with cut-offs strategically positioned to achieve balanced effect size across the ordinal categories. Finally, we assessed the discriminative ability of different electrocardiogram (ECG) characteristics using the AUC.

#### Step 2: Point-Integrated Multivariable Logistic Regression Models

We examined the individual predictive capability of the present cardiovascular component for 30-day mortality using multivariable logistic regression models that incorporated all six SOFA components. The cardiovascular SOFA component was then systematically substituted with each new variable's point group, and the independent predictive capability of each was analysed.

#### Step 3: Extending the SOFA Score

The reference SOFA score was extended by adding the new point groups in different combinations. Different effect weightings were created by doubling or tripling the assigned points thereby increasing a variable's relative weight within the experimental model. Improved discrimination of 30-day mortality by the different cardiac-extended SOFA models compared to the reference SOFA score was evaluated using AUC. Pairwise statistical comparisons of AUCs for different models were conducted using the DeLong test.^27^ The Net Reclassification Improvement (NRI) index was calculated to assess the improved discriminative performance of the four cardiac-extended SOFA score models with the highest AUC compared with the reference SOFA score.^28,29^

#### Step 4: Internal Validation of CE-SOFA in the Test Cohort

The best-performing cardiac-extended model (CE-SOFA) was selected for testing in the test cohort and its improved discriminatory performance was evaluated using both the DeLong test and NRI index. The calibration of the CE-SOFA model was assessed by plotting the observed proportion of events against the predicted mean probabilities of the calculated CE-SOFA points divided into quintiles. Goodness of fit was tested using the Hosmer-Lemeshow test.^30^

Two-sided *P* values <.05 were considered statistically significant. IBM SPSS Statistics ver. 27.0 software (IBM, Armonk, NY) and R-4.2.2 were used for statistical analyses. This study is reported in accordance with the TRIPOD data statement on reporting guidelines for a prediction model study.

## Results

### Baseline Characteristics

The 30-day mortality rates were 32% (n = 80) in the training cohort and 30% (n = 77) in the test cohort. ICU mortality rates were 32% in the training cohort (n = 79) and 31% (n = 78) in the test cohort. Non-survivors in both cohorts were older, with a greater baseline burden of comorbidities and higher severity of illness, as indicated by higher SOFA and APACHE II scores, higher cardiac biomarker levels and higher incidence of acute renal failure (Table 1). Additionally, the prevalence of pre-existing and incidence of new-onset atrial fibrillation (AF) were higher among non-survivors in both cohorts.

**Table 1.** Baseline Characteristics.

	Training cohort			Test cohort		
	Survivors (n = 170)	Non-Survivors (n = 80)	*P-*value	Survivors (n = 176)	Non-Survivors (n = 77)	*P-*value
**Demographics, no. (%)**						
Age (y), median (IQR)	68 (55-75)	76 (69-84)	<.001	72 (64-80)	80 (70-84)	<.001
Female, no. (%)	61 (36)	22 (28)	.242	69 (39)	34 (44)	.549
Smoker	99 (58)	42 (53)	.474	105 (60)	39 (51)	.233
BMI (kg/m)^b^ median (IQR)	25 (22-29)	25 (22-30)	.462	25 (22-30)	26 (23-30)	.425
CFS, median (IQR)	4 (3-5)	5 (3-6)	.001	4 (3-6)	5 (4-6)	<.001
**Comorbid history, no (%)**						
Myocardial infarction	18 (11)	13 (16)	.289	26 (15)	17 (22)	.214
Heart failure	27 (16)	30 (38)	<.001	53 (30)	30 (39)	.217
Hypertension	95 (56)	54 (68)	.108	104 (59)	54 (70)	.127
Atrial fibrillation	35 (21)	34 (43)	<.001	56 (32)	29 (38)	.006
Stroke/TIA	23 (14)	13 (16)	.705	30 (17)	11 (14)	.717
Diabetes mellitus	44 (26)	28 (35)	.182	51 (29)	23 (30)	.999
Habitual eGFR (mL/min/1.73m)^b^	69 ± 26	54 ± 25	<.001	61 ± 20	52 ± 24	.004
Liver cirrhosis	4 (2)	3 (4)	.831	2 (1)	4 (5)	.133
Cancer (previous and current)	48 (28)	23 (29)	.999	60 (34)	24 (31)	.757
CCI, median (IQR)	4 (2-6)	6 (4-7)	<.001	5 (3-6)	6 (4-8)	.003
**Hospital course, mean ± SD or median (IQR)**						
SOFA score^a^	8 ± 3	9 ± 3	<.001	8 ± 2	9 ± 3	<.001
APACHE II score	21 ± 8	27 ± 8	<.001	20 ± 7	28 ± 8	<.001
i.v. fluid (L) prior to NE	3.0 (2.0-3.5)	2.0 (1.9-3.0)	.025	3.0 (2.0-3.5)	2.0 (2.0-3.5)	.076
Duration of NE infusion (hrs)	42 (26-82)	40 (17-75)	.223	40 (19-68)	40 (19-76)	.790
Peak NE dose (µg/kg/min)	0.19 (0.1-0.34)	0.25 (0.14-0.46)	.003	0.12 (0.07-0.3)	0.25 (0.12-0.5)	<.001
Length of IMCU/ICU stay (days)	4 (2-8)	3 (2-6)	.026	4 (2-7)	2 (1-5)	<.001
hs-cTnT (ng/L)	42 (19-98)	90 (44-254)	<.001	52 (31-101)	73 (51-168)	<.001
NT-proBNP (ng/L)	4200 (1458-9548)	10550 (3545-26700)	<.001	5185 (1620-15875)	7650 (2630-23050)	.019
Highest lactate (mmol/L)	3.8 (2.2-5.9)	4.3 (2.4-8.1)	.076	3.4 (1.8-5.2)	4.4 (2.5-9.9)	<.001
CRP (mg/L), mean ± SD	255 ± 132	196 ± 122	<.001	230 ± 121	205 ± 126	.139
Highest creatinine (µmol/L)	171 (108-280)	214 (157-274)	.005	166 (113-251)	225 (171-313)	<.001
Hematocrit (%)	35 ± 6	35 ± 9	.707	34 ± 6	36 ± 8	.043
**Hospital course, no (%)**						
Normal ECG reading, (n = 221)^c^	56 (33)	6 (8)	<.001	39 (22)	13 (17)	.432
Acute renal failure^d^	106 (62)	61 (76)	.042	118 (67)	57 (74)	.338
Atrial fibrillation, new-onset	30 (18)	21 (26)	<.001	24 (14)	21 (27)	.006
Septic shock (Sepsis-3 criteria)	149 (88)	73 (91)	.530	153 (87)	70 (91)	.491
Positive blood culture	101 (59)	42 (53)	.372	112 (64)	47 (61)	.801

Abbreviations: BMI, body mass index; CRP, C-reactive protein; ECG, electrocardiography; eGFR, estimated glomerular filtration rate; hs-cTnT, high-sensitive cardiac troponin T; i.v., intravenous; ICU, intensive care unit; IMCU, intermediate care unit; IQR, interquartile range; MAP, mean arterial pressure; NE, norepinephrine; NT-proBNP, N-terminal-pro B-type natriuretic peptide; SD, standard deviation; TIA, transient ischaemic attack. APACHE II, Acute Physiology and Chronic Health Evaluation II: a severity of disease classification system calculated within 24 h of patient admission into the ICU. It incorporates 12 admission physiologic variables, the patient's age, and chronic comorbidities. 0-71 points. CCI, Charlson Comorbidity Index (age-adjusted): total score is derived by summing the assigned weights of age and 17 comorbid conditions, higher scores indicate a more severe condition with worse prognosis. 0-27 points. CFS, Clinical Frailty Scale: a judgement-based scale evaluating comorbidity-related symptoms, cognition and disability ranging from 1 (very fit) to 9 (terminally ill). SOFA score, Sequential Organ Failure Assessment score: 0-24 points where 0-4 points are assigned according to the degree of acute hematologic, hepatic, respiratory, neurologic, cardiovascular, and renal dysfunction. A greater score corresponds to greater severity.

^a^The reference score calculated upon admission to the IMCU/ICU.

^b^Registered upon admission to the IMCU/ICU.

^c^Recorded within eight hours from the point of sepsis onset.

^d^According to the Kidney Disease: Improving Global Outcomes (KDIGO) staging criteria where acute kidney injury is defined as an increase in serum creatinine >1.5 times the baseline value.

### Step 1: Cardiovascular Variable Selection and Transformation

**Figure 1.**
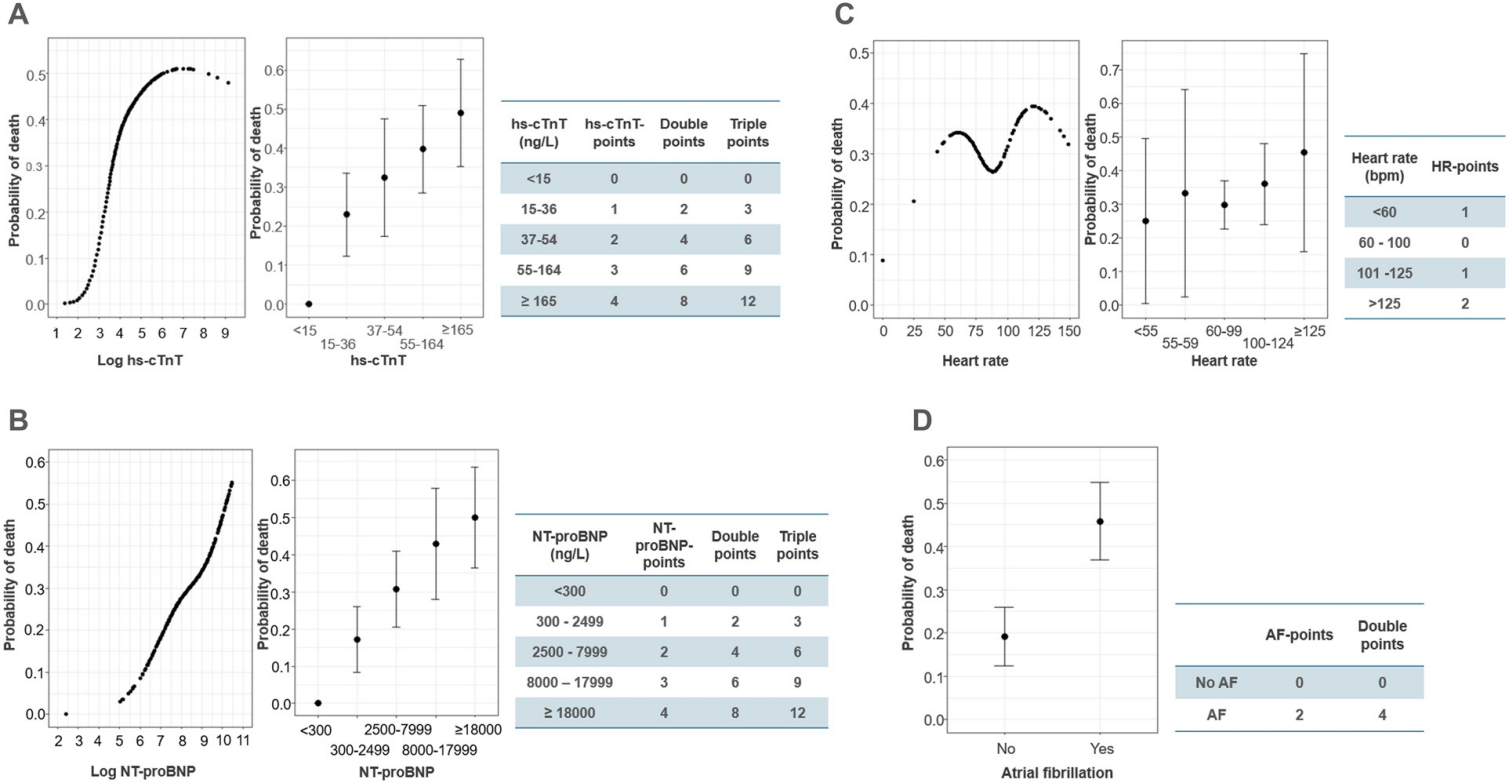
Transforming the cardiovascular variables into ordinal variables. The left panels of *A-C* show the relationship between: (A) log hs-cTnT, (B) log NT-proBNP and (C) heart rate (HR) and 30-day mortality using natural cubic spline. The middle panels of *A-C* show the mean probability of death at 30 days with 95% Confidence Interval (CI) for each ordinal category (0-4 points). The right panels of *A-C* display the calculated value cut-offs and corresponding single, double and triple weighted points. (D) The left panel shows the mean risk estimate with 95% CI for atrial fibrillation (AF), while the right panel shows the assigned single and double *AF-points*.

Figure 1 shows the natural cubic spline curves modelling the predictive ability for 30-day mortality by log hs-TnT- and log NT-proBNP level. Both cardiac biomarkers displayed a strong association between increasingly elevated plasma level and increased 30-day mortality and were thus selected for further analysis.

The natural cubic spline curves modelling the relationship between 30-day mortality and SBP, DBP, MAP, HR, PP, RPP and PPP are shown in Supplementary Figure S2. Discriminative ability in all cases was poor (AUC 0.53-0.57). We also analysed the association between the SBP, DBP, HR, PP, RPP and PPP and 30-day mortality at (i) the point of sepsis onset and (ii) on day 2 at 6 am and found no association (Supplementary Figure S2). The decision to retain HR for further analysis was made based on its status as a routine clinical vital sign, and to enable further exploration of an alternative physiological variable to MAP.

Among the ECG characteristics, only sinus rhythm and AF exhibited 30-day mortality discriminative ability, achieving AUCs of 0.63 and 0.61, respectively (Supplementary Figure S3). Given the clinical significance and predictive power of AF, we decided to include it as a variable for further analysis. Pre-existing and new-onset AF were combined into a single variable in our analyses for several reasons: both types are linked to increased morbidity and mortality, and both reflect cardiovascular instability.^31–33^ Additionally, using a single variable simplifies the scoring process and aligns with clinical practice, where management of AF is similar regardless of onset timing.

Hs-cTnT, NT-proBNP and HR were transformed into ordinal variables. The final cut-off values and the resultant *hs-cTnT-points, NT-proBNP-points* and *HR-points* are presented in Figure 1. The presence of AF was transformed into a binary variable, with *AF-points* assigned as follows: patients without AF (0 points), patients with pre-existing or new-onset AF (2 points).

### Step 2: Point-Integrated Multivariable Logistic Regression Models

After adjustment for the other five SOFA score components, the *hs-cTnT-points, NT-proBNP-points,* and *AF-points* were associated with 30-day mortality, while the current cardiovascular component and *HR-points* were not (Table 2). Furthermore, the *hs-cTnT-points, NT-proBNP-points* and *AF-points* displayed a stronger independent association with mortality compared to the other five SOFA components. These findings remained consistent even when the potential confounders, age, and sex, were included in the regression models (Supplementary Table S1).

**Table 2.** Integrating the selected cardiovascular variables into the SOFA score in the training cohort. Multivariable logistic regression analysis of the six different SOFA score components and their association with 30-day mortality with: (i) the current cardiovascular SOFA component. The cardiovascular SOFA component was then substituted with (ii) the *hs-cTnT-points*; (iii) the *NT-proBNP-points*; (iv) the *AF-points* and (v) the *HR-points*.

	(i)		(ii)		(iii)		(iv)		(v)	
	OR (95% CI)	*P*-value	OR (95% CI)	*P*-value	OR (95% CI)	*P*-value	OR (95% CI)	*P*-value	OR (95% CI)	*P*-value
Neurological SOFA (0-4p)	1.39 (1.09-1.77)	0.008	1.33 (1.02-1.72)	.033	1.38 (1.07-1.78)	.012	1.46 (1.13-1.89)	.004	1.43 (1.12-1.84)	.005
Respiratory SOFA (0-4p)	1.27 (0.96-1.68)	0.091	1.32 (0.98-1.76)	.066	1.21 (0.91-1.60)	.197	1.27 (0.95-1.70)	.106	1.30 (0.97-1.73)	.075
Renal SOFA (0-4p)	1.27 (1.00-1.60)	0.049	1.06 (0.82-1.38)	.637	1.06 (0.82-1.38)	.652	1.20 (0.94-1.54)	.143	1.24 (0.98-1.57)	.080
Hepatic SOFA (0-4p)	1.43 (1.02-2.01)	0.036	1.72 (1.20-2.47)	.003	1.39 (0.98-1.96)	.039	1.47 (1.03-2.09)	.032	1.48 (1.05-2.09)	.024
Coagulation SOFA (0-4p)	1.29 (0.96-1.73)	0.091	1.36 (1.00-1.86)	.049	1.38 (1.02-1.86)	.039	1.37 (0.99-1.87)	.051	1.28 (0.96-1.72)	.096
Cardiovascular SOFA (0-4p)	0.85 (0.62-1.17)	0.320	—	—	—	—	—	—	—	—
hs-cTnT-points (0-4 p)	—	—	1.88 (1.43-2.46)	<.001	—	—	—	—	—	—
NT-proBNP-points (0-4 p)	—	—	—	—	1.68 (1.28-2.21)	<.001	—	—	—	—
AF-points (0 or 2p)		—	—	—	—	—	4.15 (2.24-7.70)	<.001	—	—
HR-points (0-2p)	—	—	—	—	—	—	—	—	0.91 (0.61-1.34)	.623

Abbreviations: AF, atrial fibrillation; CI, confidence interval; hs-cTnT, high-sensitive cardiac troponin T; HR, heart rate; NT-proBNP, N-terminal-pro B-type natriuretic peptide; OR, odds ratio, SOFA, Sequential Organ Failure Assessment.

### Step 3: Extending the SOFA Score

The AUC for each new point group added to the SOFA score was calculated (Supplementary Figure S4). Upon pairwise comparison of the AUCs using the DeLong test, the 30-day mortality discrimination ability of the SOFA score significantly improved with the addition of the *hs-cTnT-points* (*P *< .001), *NT-proBNP-points* (*P *= .005) or *AF-points* (*P *< .001) but not with the *HR-points* (*P *= .929). To explore whether AF and HR could act as covariates, their relationship with 30-day mortality was tested in the same logistic regression model. AF was associated with 30-day mortality (OR 1.9, *P* < .001), whereas HR (OR 1.1, *P* = .674) was not. No further analysis was conducted using the *HR-points*.

The extended SOFA score was further improved by varying the relative weights of the *hs-cTnT-, NT-proBNP- or AF-points* (Supplementary Figure S5). All extended SOFA score models, featuring differently weighted combinations of single, double, or triple *hs-cTnT-, NT-proBNP- or AF-points* significantly improved the prediction of 30-day mortality compared to the SOFA score alone (Supplementary Figures S6, S7 and S8).

**Figure 2.**
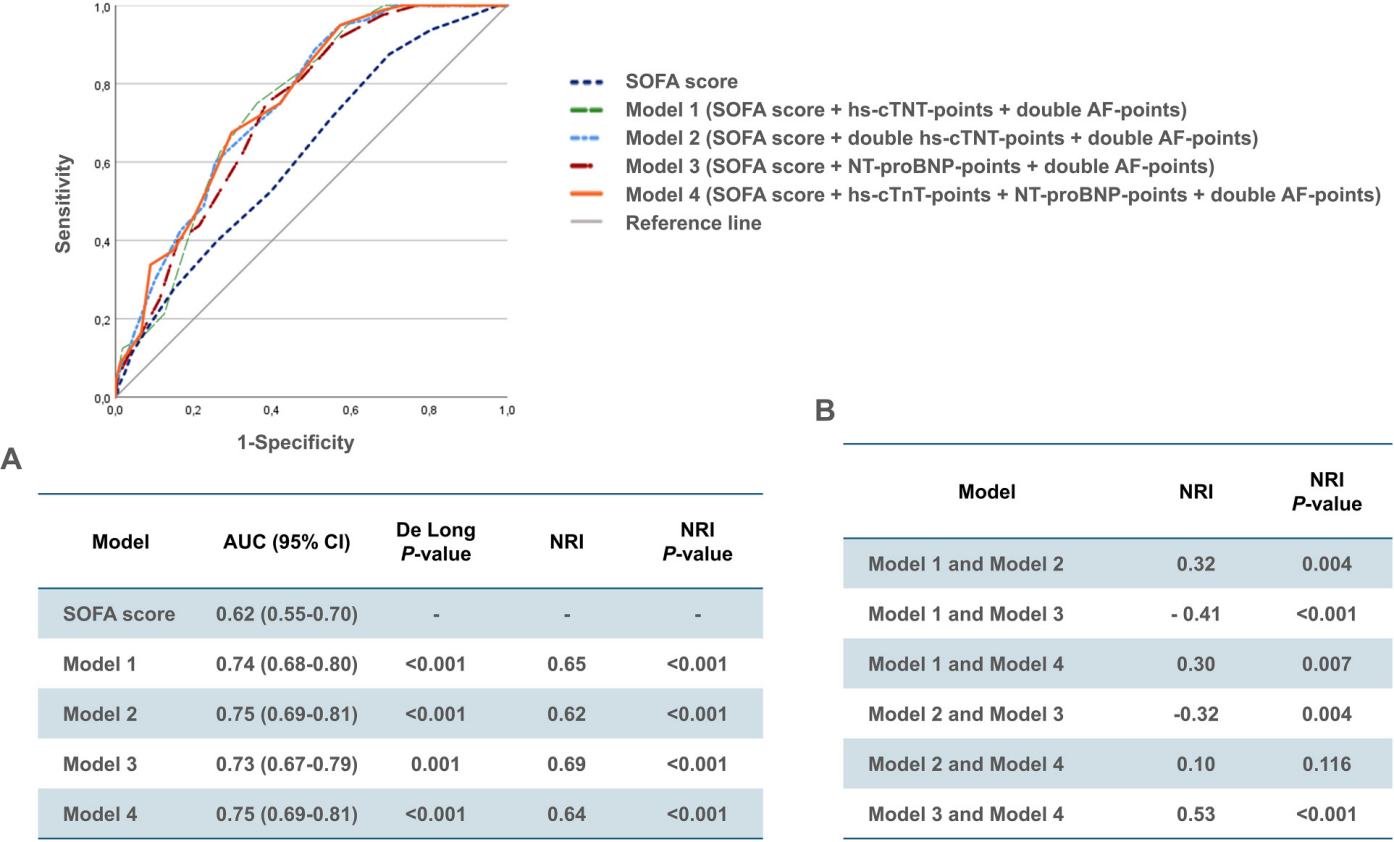
Receiver operating curves with (A) corresponding area under the curve (AUC) and 95% confidence interval (CI) for the four best-performing cardiac-extended SOFA score models, with results of the DeLong test and estimated net reclassification improvement (NRI) index in the training cohort. (B) Comparison of the estimated discriminative ability among each of the four cardiac-extended SOFA score models in the training cohort using the NRI index method.

Finally, four extended SOFA score models, with an AUC ranging from 0.73 to 0.75 were selected (Models 1-4; Figure 2). Upon pair-wise comparison using the DeLong test and NRI index, all four models displayed improved discriminative ability for 30-day mortality compared to the SOFA score alone (Figure 2A). Furthermore, statistical significance was evident in the NRI calculations among the four combined models in all cases except between Model 2 and Model 4 (Figure 2B). Model 4, comprising the SOFA score + *hs-cTnT-points + NT-proBNP-points + *double *AF-points*, was chosen as the final CE-SOFA model.

### Step 4: Internal Validation of CE-SOFA in the Test Cohort

**Figure 3.**
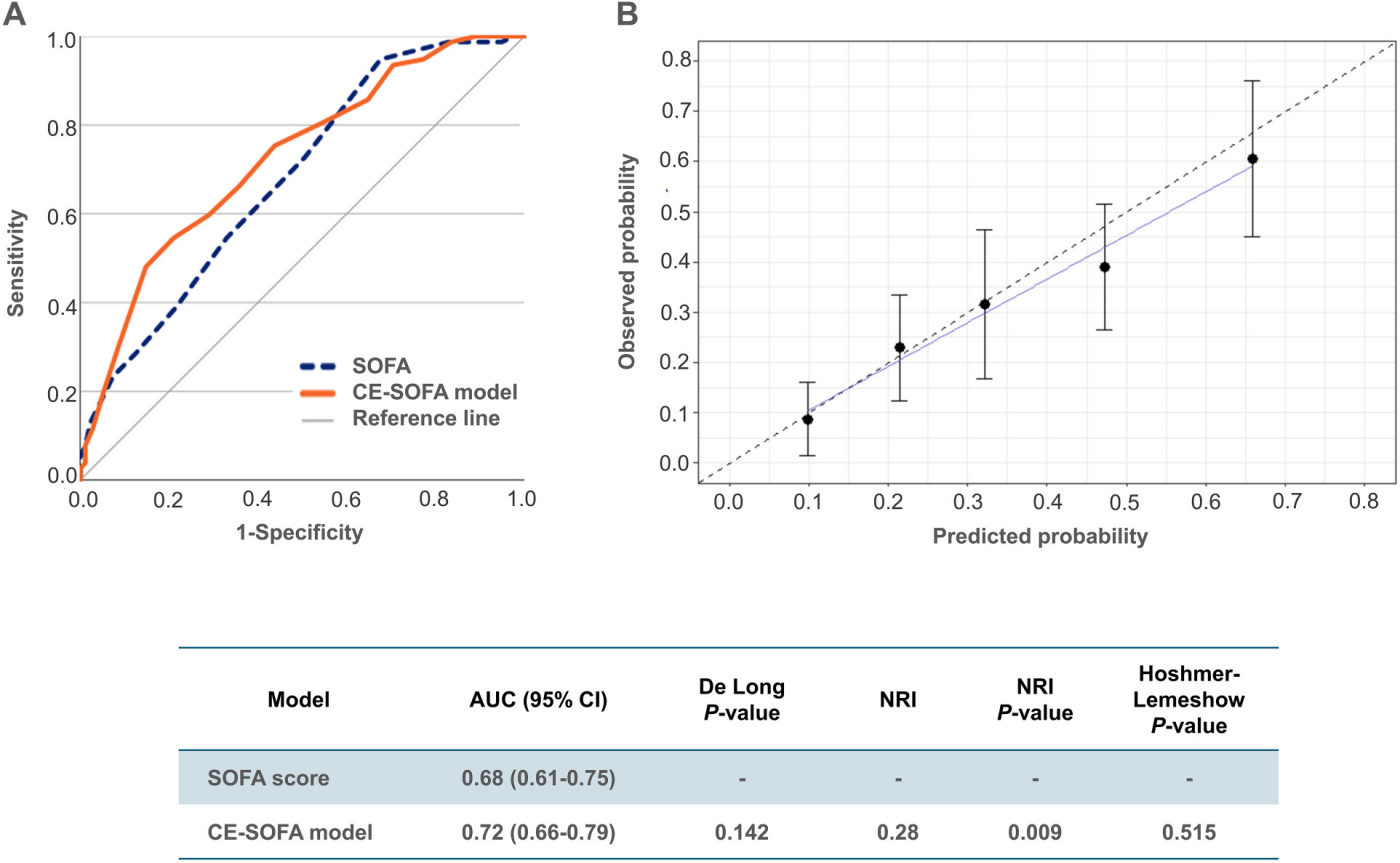
Validation of the cardiac-extended SOFA (CE-SOFA) model in the test cohort. (A) Receiver operating curves with the corresponding area under the curve (AUC) and 95% Confidence Interval (CI) for the SOFA score and the final CE-SOFA model, with the results of the DeLong test and estimated Net Reclassification Improvement (NRI) index. The CE-SOFA model (0-36 points) comprises the sum of the SOFA score (0-24 points), *hs-cTnT-points* (0-4 points), *NT-proBNP-points* (0-4 points), and double *AF-points* (0 or 4 points). (B) Calibration of the CE-SOFA model, reflecting the accuracy with which the new model estimates absolute risk. The error bars around the calibration curve indicate the confidence intervals. The Hoshmer-Lemeshow test evaluates the goodness of fit between the predicted probability of 30-day mortality by the CE-SOFA model divided into quintiles, and the observed probability; a small *P*-value suggests poor goodness of fit.

In the test cohort, the AUC for the SOFA score (AUC 0.68) exceeded that of the training cohort (AUC 0.62). The CE-SOFA model exhibited significantly greater discriminative ability than the SOFA score according to the NRI index, though not confirmed by the DeLong test (Figure 3). Calibration of the CE-SOFA model was good (Figure 3B), and the Hosmer-Lemeshow test affirmed a high goodness of fit. Supplementary Table S2 contains the CE-SOFA model parameters integrated into the current SOFA score. Supplementary Figure S9 and Figure S10 depict the distribution of the SOFA score and the CE-SOFA model stratified for 30-day mortality in both cohorts.

## Discussion

This study aimed to augment the existing cardiovascular SOFA component enabling the detection of myocardial injury and dysfunction, and to improve the discriminatory power of the composite SOFA score for 30-day mortality. We assessed ten widely available and simple variables indicative of cardiovascular dysfunction in a cohort of critically ill sepsis patients requiring vasopressor support. While all physiological variables, including MAP and HR, proved to be poor predictors of 30-day mortality, the presence of AF, along with measured levels of hs-cTnT and NT-proBNP demonstrated individual discriminatory abilities comparable to the composite SOFA score itself. The CE-SOFA model, comprising the sum of the SOFA score (with points for MAP +/- vasoactive agent), *hs-cTnT-points, NT-proBNP-points,* and double *AF-points,* can assess four aspects of cardiovascular dysfunction: hemodynamic instability, acute myocardial injury, myocardial dysfunction and arrythmia, respectively. In the test cohort, CE-SOFA showed promising results, demonstrating good calibration, and improved discriminatory ability for 30-day mortality using AUC and NRI index measures.

The ability to promptly initiate and sustain an effective acute cardiovascular response, ensuring adequate organ perfusion, is pivotal to the hospital course and survival of critically ill patients. Since the introduction of the SOFA score, studies have revealed that cardiovascular comorbidities are overrepresented in sepsis patients, with up to 70% of those with septic shock experiencing concurrent myocardial dysfunction.^34,35^ Recent reports have also highlighted a notable incidence of sepsis-related myocardial infarction and/or cardiogenic shock.^36–38^ Myocardial dysfunction in cardiovascular failure cannot be discerned through MAP measurements alone; additional assessment using visual techniques or cardiac biomarkers are often required to differentiate it from pathological vasodilation and vasoplegia. Not all hospitals have readily available trained medical professionals capable of performing acute echocardiography or point of care ultrasound. However, cardiac biomarkers are now widely used, offering the advantage of longitudinal monitoring and the potential to guide personalized investigation and treatment.

Recent publications have highlighted that the current cardiovascular SOFA component, which relies on MAP measurements and the administration of vasoactive agents, may no longer be as clinically relevant as it once was.^9,11–13,17^ As the approach to septic and cardiogenic shock therapy evolves towards individualization, the predetermined allocation of cardiovascular points for vasoactive agent administration may become increasingly inappropriate.^14,15^ Furthermore, while maintaining an adequate MAP to ensure perfusion pressure is deeply rooted in clinical practice, an optimal “target” MAP remains undetermined.^39^ Studies have reported conflicting findings regarding the benefits of higher versus lower targets, underscoring the significance of considering individual patient factors, such as chronic arterial hypertension, when determining the optimal MAP.^40–42^ Importantly, trials have shown that adhering to MAP resuscitation targets does not always ensure adequate regional blood flow and microcirculatory perfusion.^43,44^

In this study, we illustrate that extending the current MAP-based cardiovascular SOFA component with assigned points for cardiac biomarker levels improves its discriminative ability. The internationally recommended cardiac biomarkers hs-cTnT and NT-proBNP are extensively researched, validated, and widely implemented assays used for assessing acute myocardial injury and dysfunction, respectively.^26,45,46^ These biomarkers have become integral in evaluating cardiovascular risk across various clinical contexts beyond myocardial infarction.^26,45,47,48^ Numerous studies have underscored the strong correlation between increasing levels of both biomarkers and the severity of myocardial injury/dysfunction, as well as higher mortality, irrespective of the underlying condition.^18,26,45,49–52^ Furthermore, both cardiac biomarkers are used clinically to track myocardial injury and dysfunction over time which is an integral characteristic of the variables making up each component of the SOFA score.^3,26,45^

In line with the performance by the cardiac biomarkers, the addition of points for the presence of AF further improved the prognostic discrimination of the composite SOFA-score for short-term mortality in all our exploratory models. AF, the most prevalent cardiac arrhythmia in critically ill patients, is a well-established risk factor for adverse outcomes, including prolonged hospital stays, cerebrovascular events and mortality.^31,53–55^ Furthermore, the incidence of new-onset AF, affecting around 14% of ICU patients, increases with illness severity.^56,57^ The clinically adverse cardiovascular effects of AF, such as irregular heart rate, tachycardia, loss of atrioventricular synchrony, and potential underlying ventricular dysfunction, can all contribute to myocardial dysfunction and adversely affect central hemodynamic status.^58,59^ Our findings endorse its consideration for incorporation into a future cardiovascular SOFA component.

While the SOFA score provides valuable insight into the extent of different organ dysfunction, its design does not allow the differentiation of distinct sub-types of organ injury and failure. Elevated serum creatinine levels alone are unable to discriminate between prerenal and intrinsic renal injury. Similarly, elevated cardiac biomarker levels, despite correlating with severity of injury and dysfunction, often stem from diverse causes, highlighting the need for personalized clinical investigation. Importantly, their elevation may reveal previously undetected cardiovascular diseases with potentially significant clinical, therapeutic, and prognostic implications.

It can be argued that the cardiovascular component is the most critical among the six SOFA score components, as cardiovascular dysfunction can precipitate dysfunction in all other organs. Giving greater proportional weight to the cardiovascular component in the CE-SOFA model resulted in a moderate improvement in AUC compared to the current SOFA score. However, in the context of critical care, even moderate improvements in AUC can be clinically important, as small enhancements in clinical and predictive accuracy can lead to better patient outcomes through more timely and appropriate interventions. By adding additional points to the SOFA score and incorporating more detailed information about cardiovascular function, we enhance its predictive power and enable more effective severity stratification. Future studies are needed to show if this can be translated into more effective treatment plans.

External validation in additional cohorts is necessary to substantiate our findings. This is a single-centre investigation, and local treatment practices may limit the generalizability of our results to other sepsis cohorts. Increasing the weight of a single score component can reduce generalizability and complicate the interpretation of the score for clinicians. The selection of patients is a limitation of this study. All patients in our cohort required vasopressor support at some point during their IMCU/ICU stay, which may have introduced a degree of selection bias. Therefore, we cannot be certain that our results apply to sepsis patients with less severe circulatory instability and myocardial strain. Additionally, there may be selective mortality bias, as patients who died before admission to the IMU/ICU were not included.

In the test cohort, the CE-SOFA model did not significantly improve discriminatory ability for 30-day mortality compared to the SOFA-score when assessed using the DeLong test, despite a notable difference in AUC. A plausible explanation could be that both the NRI index and DeLong test evaluate the same null hypothesis, but the DeLong test has the effect of reducing the power to detect improvement in predictive accuracy.^60^

It would have been desirable to have had repeated samples of hs-cTnT and NT-proBNP to evaluate how the two cardiac biomarkers perform over time. However, we only had two or more samples of hs-cTnT and NT-proBNP in 65% and 24% of patients in the training cohort, respectively and consequently, such an analysis could not be performed. Finally, while cardiac troponins and NT-proBNP are widely implemented biomarkers in developed countries, their routine availability in less developed countries may be limited by financial, infrastructural, and logistical challenges.

## Conclusions

The CE-SOFA model facilitates the reflection of myocardial injury and dysfunction and improves 30-day mortality discrimination compared to the SOFA score in a cohort of critically ill sepsis patients. Prospective external validation of the CE-SOFA model is the next step to further substantiate a revised cardiovascular component in a future SOFA 2.0.

## Supplemental Material

sj-docx-1-jic-10.1177_08850666241282294 - Supplemental material for Development of an Extended Cardiovascular SOFA Score Component Reflecting Cardiac Dysfunction with Improved Survival Prediction in Sepsis: An Exploratory Analysis in the Sepsis and Elevated Troponin (SET) Study Supplemental material, sj-docx-1-jic-10.1177_08850666241282294 for Development of an Extended Cardiovascular SOFA Score Component Reflecting Cardiac Dysfunction with Improved Survival Prediction in Sepsis: An Exploratory Analysis in the Sepsis and Elevated Troponin (SET) Study by S. Lörstad, Y. Wang, S. Tehrani, S. Shekarestan, P. Åstrand, P. Gille-Johnson, T. Jernberg and J. Persson in Journal of Intensive Care Medicine

## Data Availability

The datasets used and analysed during the current study are available from the corresponding author upon reasonable request.
