# Supplementary material for: Development of an Extended Cardiovascular SOFA Score Component Reflecting Cardiac Dysfunction with Improved Survival Prediction in Sepsis: An Exploratory Analysis in the Sepsis and Elevated Troponin (SET) Study
Source: J Intensive Care Med. 2024 Oct 1;40(3):320–30. doi: 10.1177/08850666241282294 (PMC11915778; doi:10.1177/08850666241282294)
Supplement: sj-docx-1-jic-10.1177_08850666241282294 - Supplemental material for Development of an Extended Cardiovascular SOFA Score Component Reflecting Cardiac Dysfunction with Improved Survival Prediction in Sepsis: An Exploratory Analysis in the Sepsis and Elevated Troponin (SET) Study [file sj-docx-1-jic-10.1177_08850666241282294.docx]

Supplementary Material

**Table S1** Integrating the four ordinal variables into the SOFA score. Multivariable analysis of the six different SOFA score components and their association with 30-day mortality with: (i) the current cardiovascular SOFA component. The current cardiovascular SOFA component was then substituted with (ii) the *hs-cTnT-points*; (iii) the *NT-proBNP-points*; (iv) the *AF-points*; (v) the *HR-points* and their association with 30-day mortality was analysed with additional adjustment for potential confounders, age and sex.

**
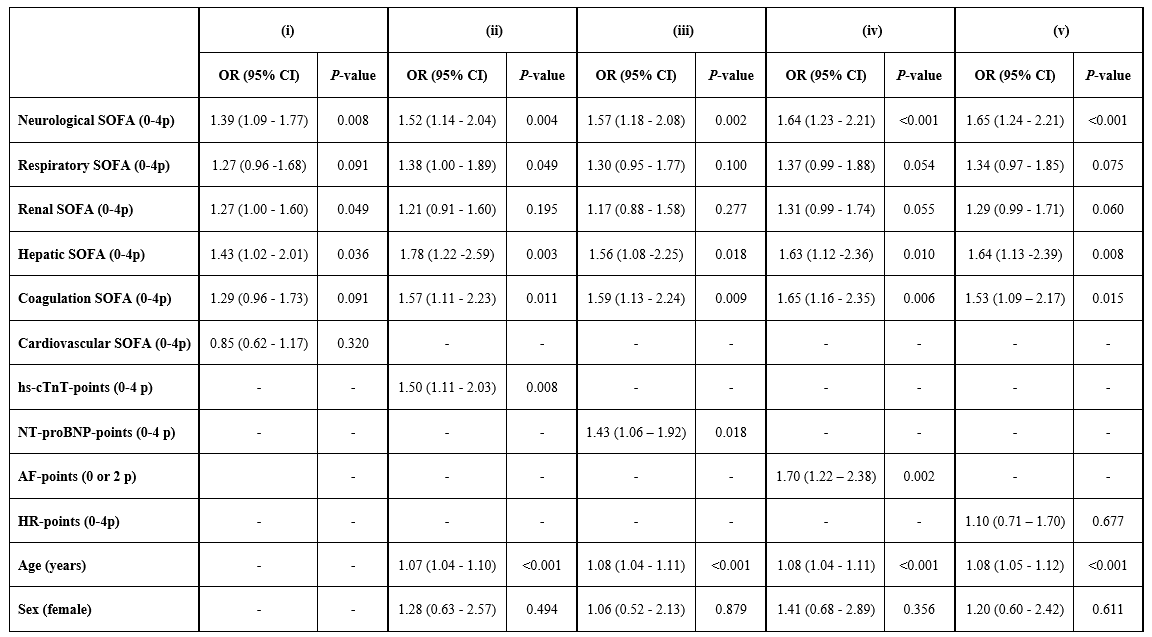
**

**Abbreviations:** CI, confidence interval; OR, odds ratio.

**Table S2** The CE-SOFA model integrated into the Sequential Organ Failure Assessment Score (adapted from Singer et al.)**[1]**

|  | | | | | |
| --- | --- | --- | --- | --- | --- |
|  | **Score** | | | | |
| **Organ system** | **0** | **1** | **2** | **3** | **4** |
| Central Nervous System |  | | | | |
| Glasgow Coma Scale | 15 | 13-14 | 10-12 | 6-9 | <6 |
| Respiration |  | | | | |
| PaO_2_/FiO_2_, (kPa) | ≥53.3 or oxygen saturation > 96% on room air | <53.3 or oxygen saturation 92-96% on room air | <40 or oxygen saturation <92% on room air | <26.7 with respiratory support | <13.3 with respiratory support |
| Cardiovascular (points assigned for MAP AND hs-cTnT AND and NT-proBNP AND presence of atrial fibrillation) | | | | | |
| MAP | MAP ≥70 mmHg | MAP <70 mmHg | Dopamine ≤5 µg/kg/min or dobutamine (any dose) to maintain MAP ≥65 mmHg | Dopamine 5-15 or noradrenaline ≤0.1 or adrenaline ≤0.1 (µg/kg/min) to maintain MAP ≥65 mmHg | Dopamine ≥15 or noradrenaline ≥0.1 or adrenaline ≥0.1 (µg/kg/min) to maintain MAP ≥65 mmHg |
| hs-cTnT (ng/L) | 0-14 | 15-36 | 37-54 | 55-164 | ≥165 |
| NT-proBNP (ng/L) | <300 | 300-2499 | 2500-7999 | 8000-17999 | ≥18000 |
| Atrial fibrillation* | No |  |  |  | Yes |
| Renal (points assigned for either creatinine level OR urine output) | | | | | |
| Creatinine (µmol/L) | <110 | 110-170 | 171-299 | 300-400 | >400 |
| Urine output (ml/day) |  |  |  | 200-500 | <200 |
| Liver |  | | | | |
| Bilirubin (µmol/L) | <20 | 20-32 | 33-101 | 102-204 | >204 |
| Coagulation |  | | | | |
| Platelets (x10^9^/µg/L) | ≤150 | <150 | <100 | <50 | <20 |
| **Abbreviations:** FiO_2_, fraction of inspired oxygen; hs-cTnT, high-sensitivity cardiac troponin T; MAP, mean arterial pressure; ; NT-proBNP, N-terminal pro B-type natriuretic peptide; PaO_2_, partial pressure of oxygen.  Glasgow Coma Scale: ranges from 3-15, higher score demonstrates better neurological function.  *4 points are assigned for both pre-existing and new-onset atrial fibrillation. | | | | | |

**Figure S1** Flow chart describing the Sepsis and Elevated Troponin (SET) study patient inclusion process (adapted from Lörstad et al.)**[2]**

**
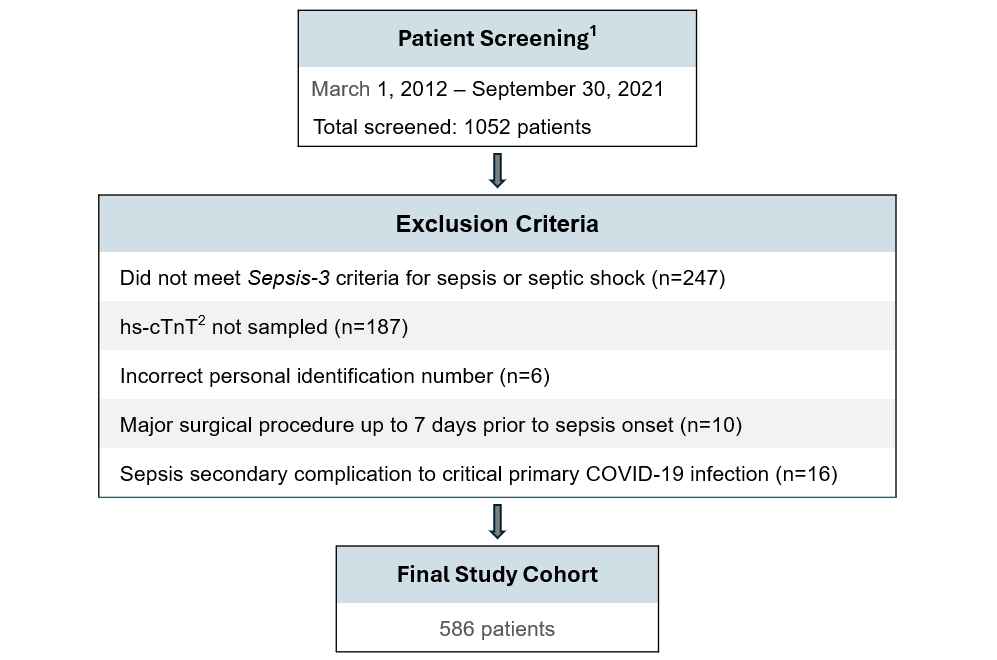
**

^1^ Patients were screened using the hospital journal system TakeCare© and the Swedish Intensive Care Register. The inclusion process involved identifying patients with specific ICD-10 codes: A40, A41, R57.2, and R65.1. Additionally, the ATC code C01CA03, which indicates norepinephrine treatment, was used to identify patients who had received norepinephrine during their hospital stay.

^2^ hs-cTnT, high-sensitivity cardiac troponin T.

**Figure S2** Natural cubic splines showing the associations between the physiological measurements: *(A)* systolic blood pressure (SBP); *(B)* diastolic blood pressure (DBP); *(C)* mean arterial pressure (MAP); *(D)* heart rate (HR); *(E)* pulse pressure (PP); *(F)* rate pressure product (RPP); *(G)* proportional pulse pressure (PPP) and 30-day mortality in the training cohort. The associations are illustrated for three different timepoints: (i) at sepsis onset, (ii) at IMCU/ICU admission, and (iii) on Day 2 at 6 am.
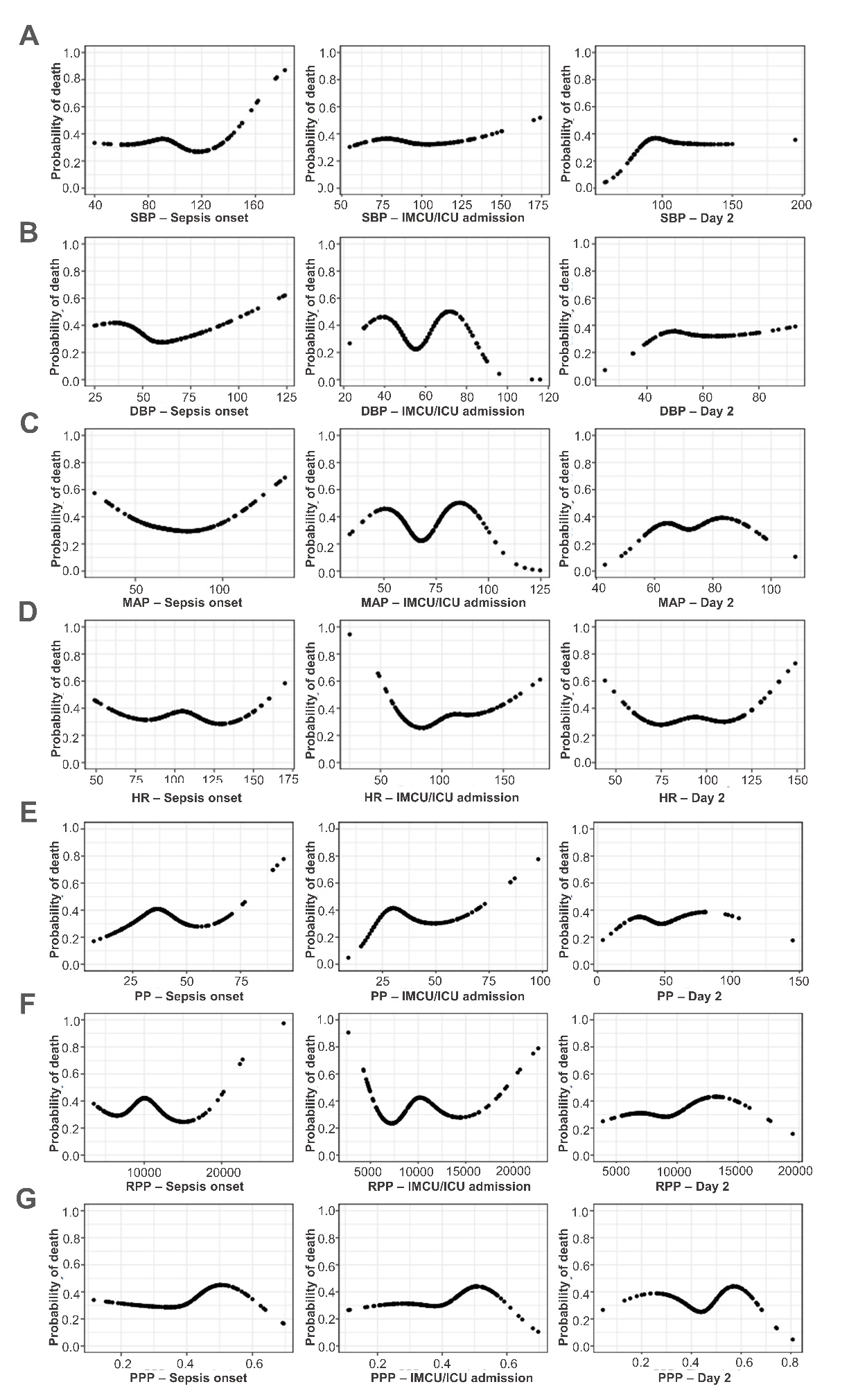


**Figure S3** Receiver operating curves with corresponding area under the curve (AUC) and 95% Confidence Interval (CI) for the different electrocardiogram characteristics and 30-day mortality in the training cohort.


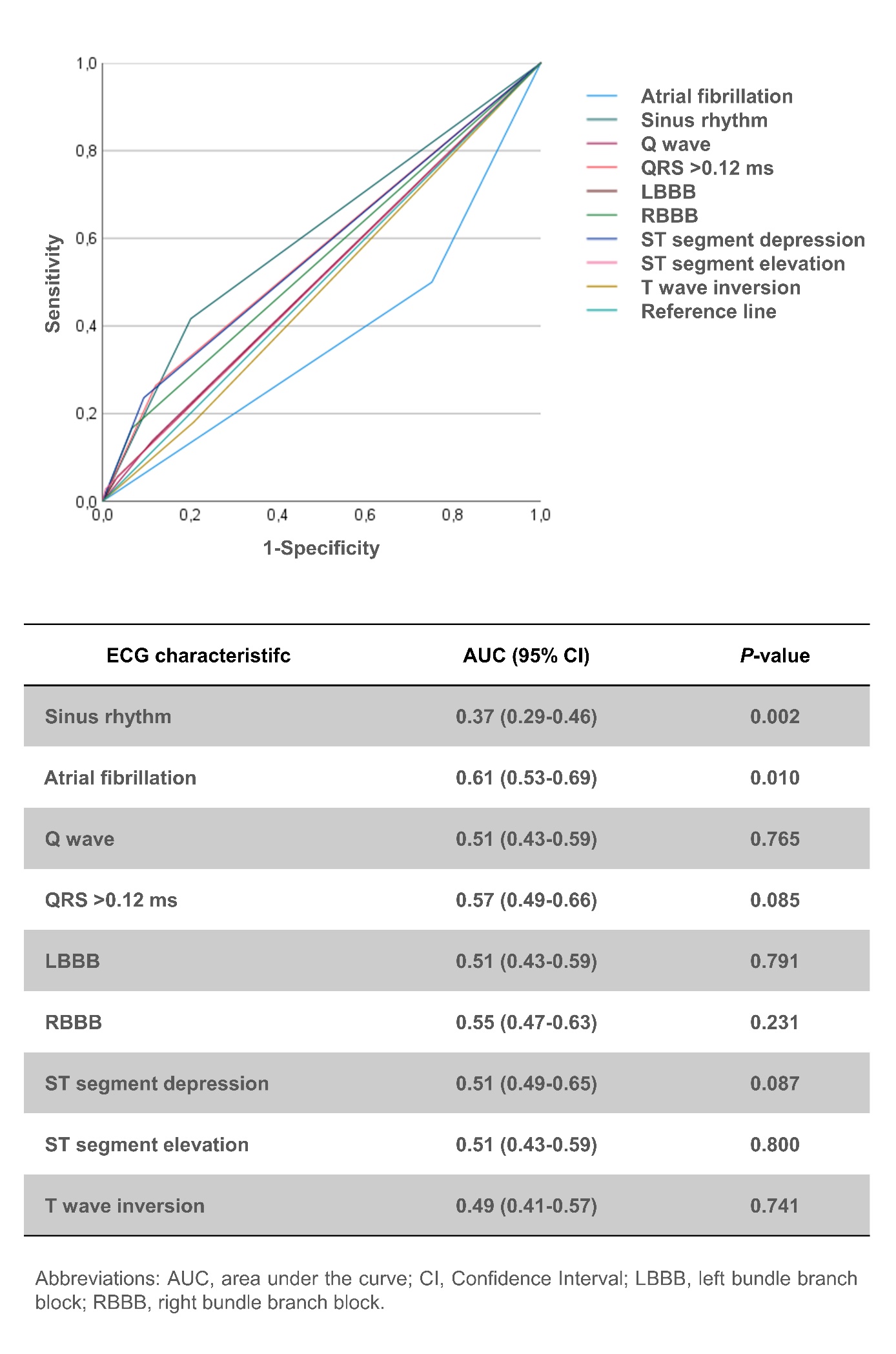


**Figure S4** Receiver operating curves with corresponding area under the curve (AUC) and 95% Confidence Interval (CI) for the SOFA score and the addition of single points for each of the ordinal variables and atrial fibrillation in the training cohort.


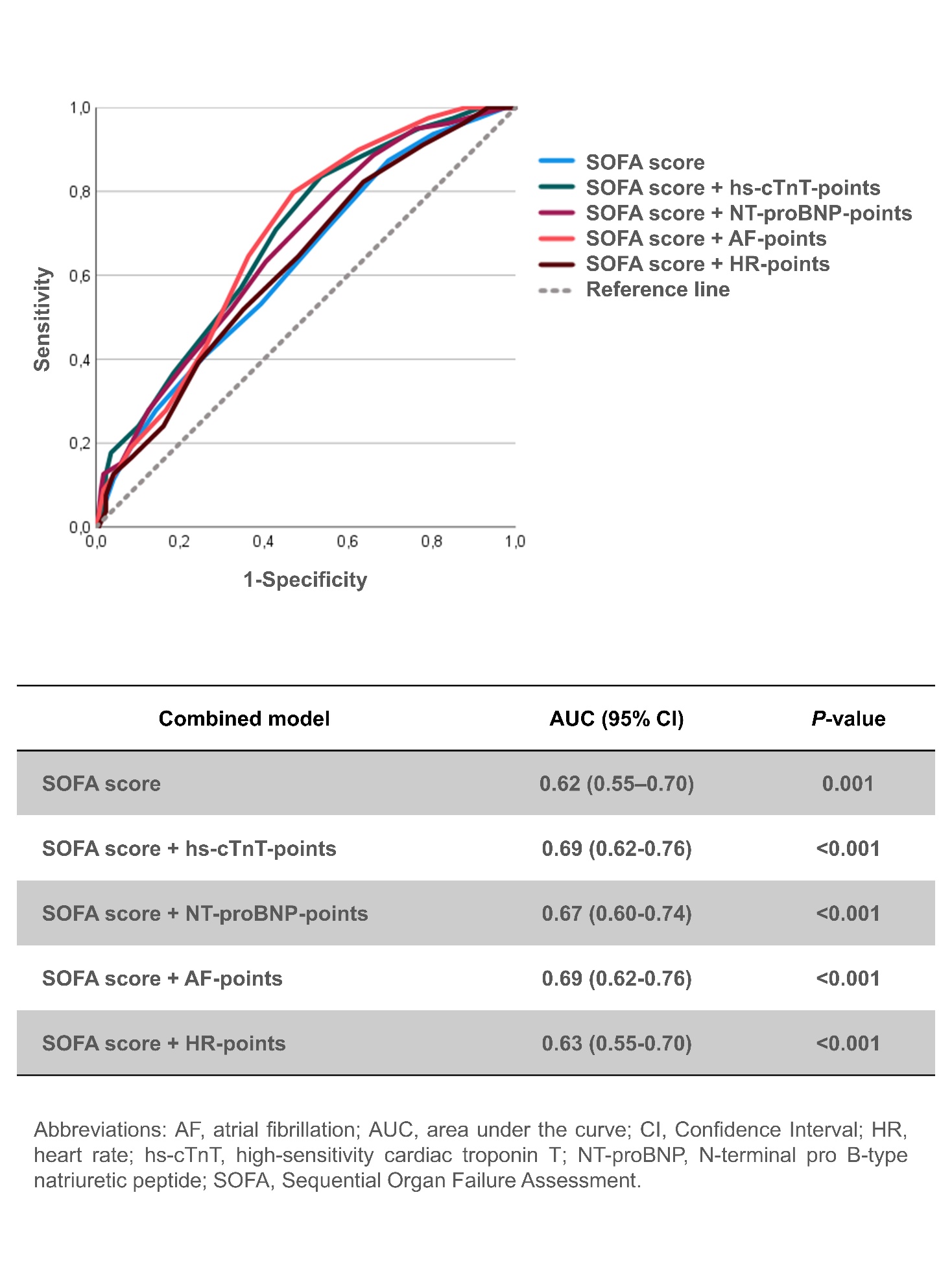


**Figure S5** Receiver operating curves with corresponding area under the curve (AUC) and 95% confidence interval (CI) for the SOFA score and the addition of double (0,2,4,6,8) and triple points (0,3,6,9,12) for the hs-cTnT and NT-proBNP variables and double points (0 and 4) for the AF variable in the training cohort.


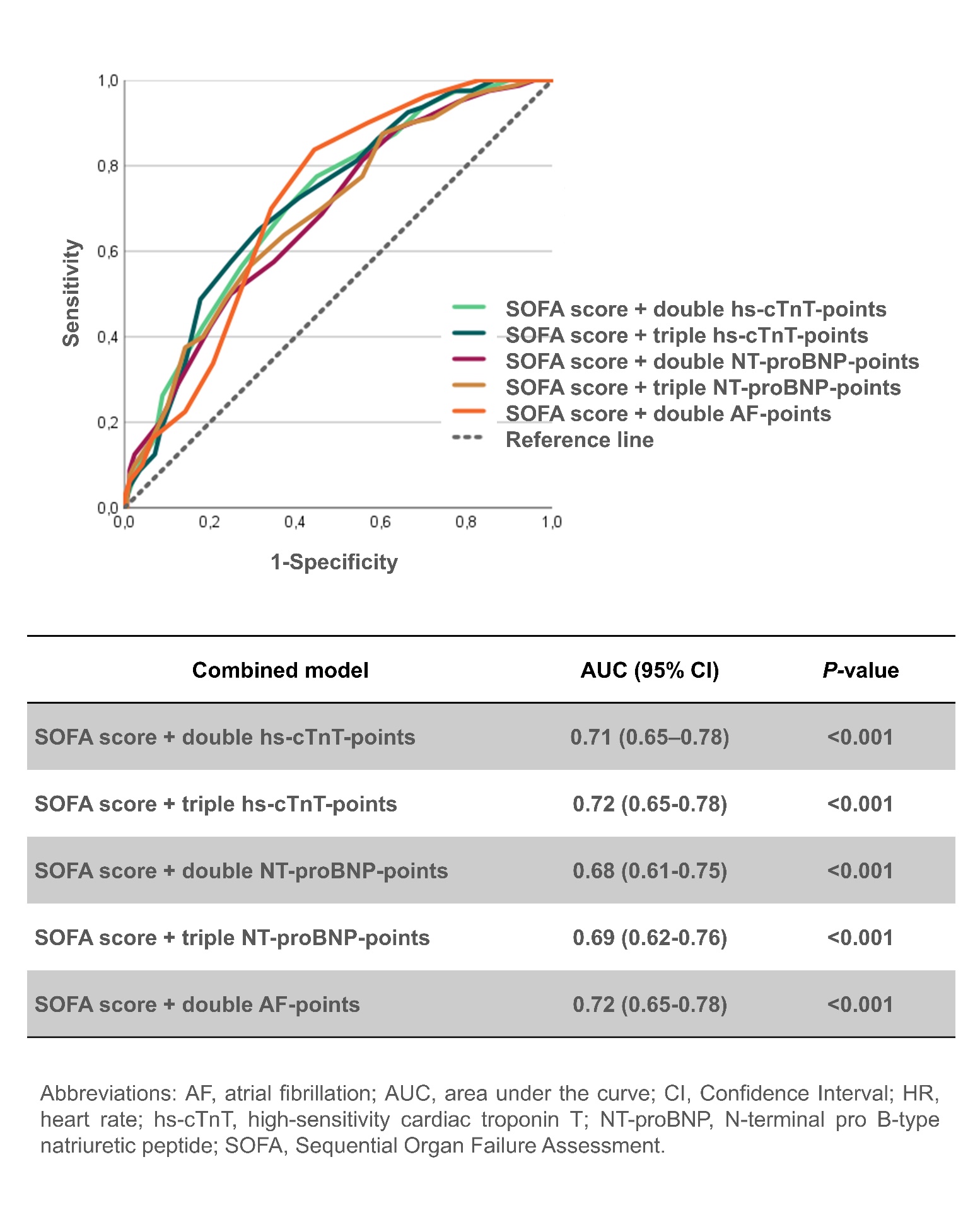


**Figure S6** Receiver operating curves with corresponding area under the curve (AUC) and 95% Confidence Interval (CI) for the SOFA score with differently weighted combinations of single and double *hs-cTnT-points* and *AF-points* in the training cohort.


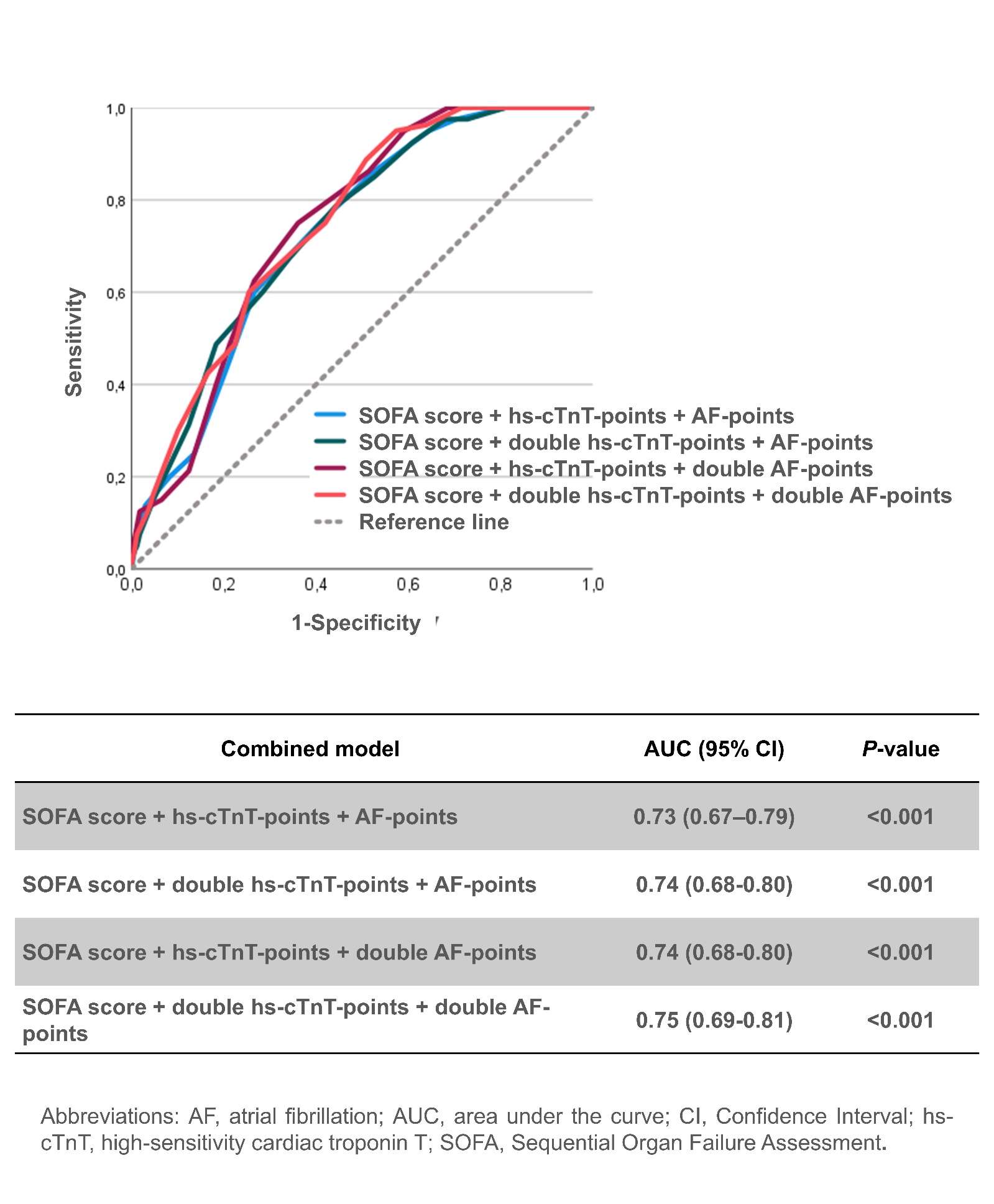


**Figure S7** Receiver operating curves with corresponding area under the curve (AUC) and 95% Confidence Interval (CI) for the SOFA score with differently weighted combinations of single and double *NT-proBNP-points* and *AF-points* in the training cohort.


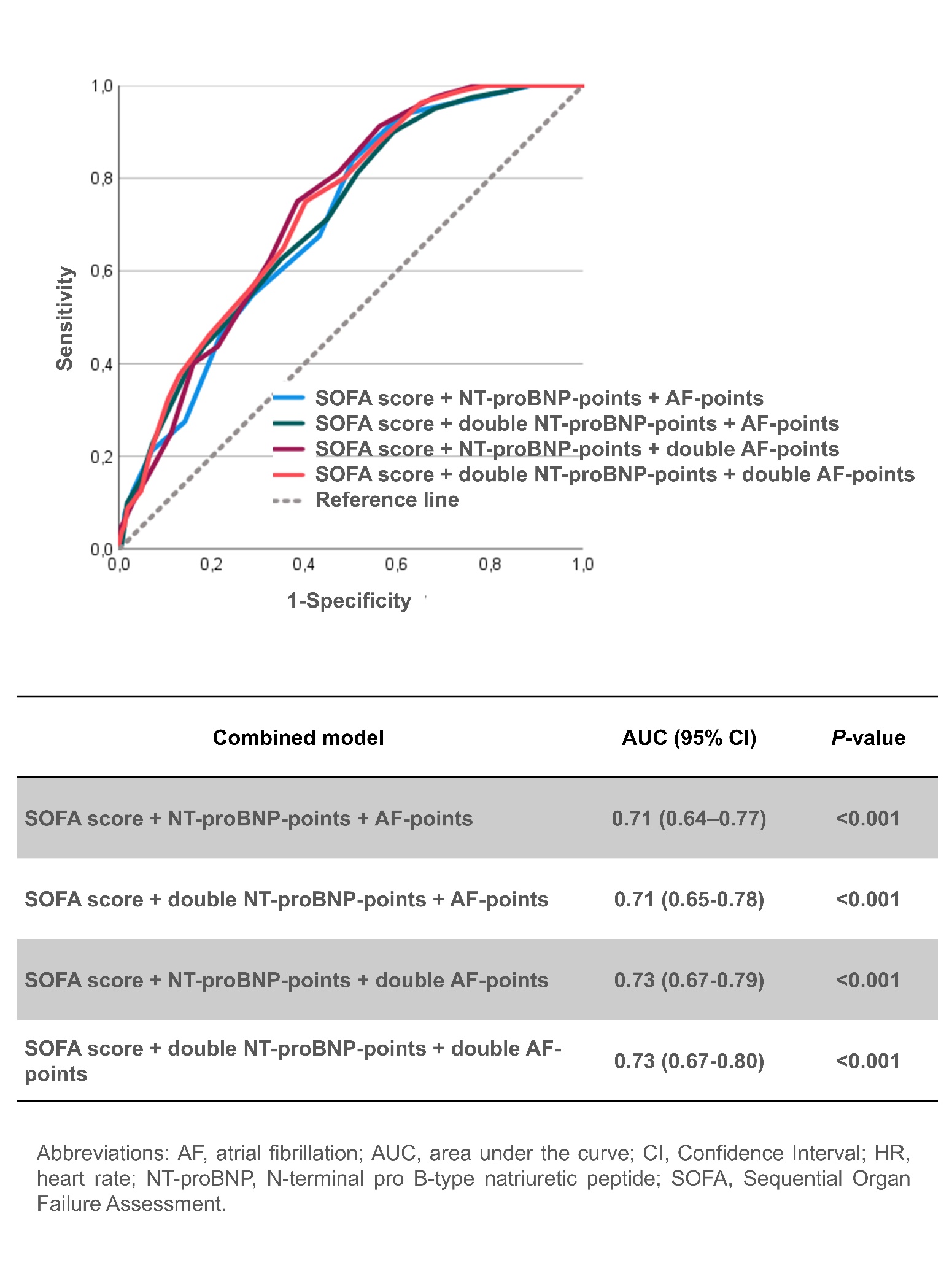


**Figure S8** Receiver operating curves with corresponding area under the curve (AUC) and 95% Confidence Interval (CI) for the SOFA score with differently weighted combinations of single and double *hs-cTnT-points*, *NT-proBNP-points* and *AF-points* in the training cohort.


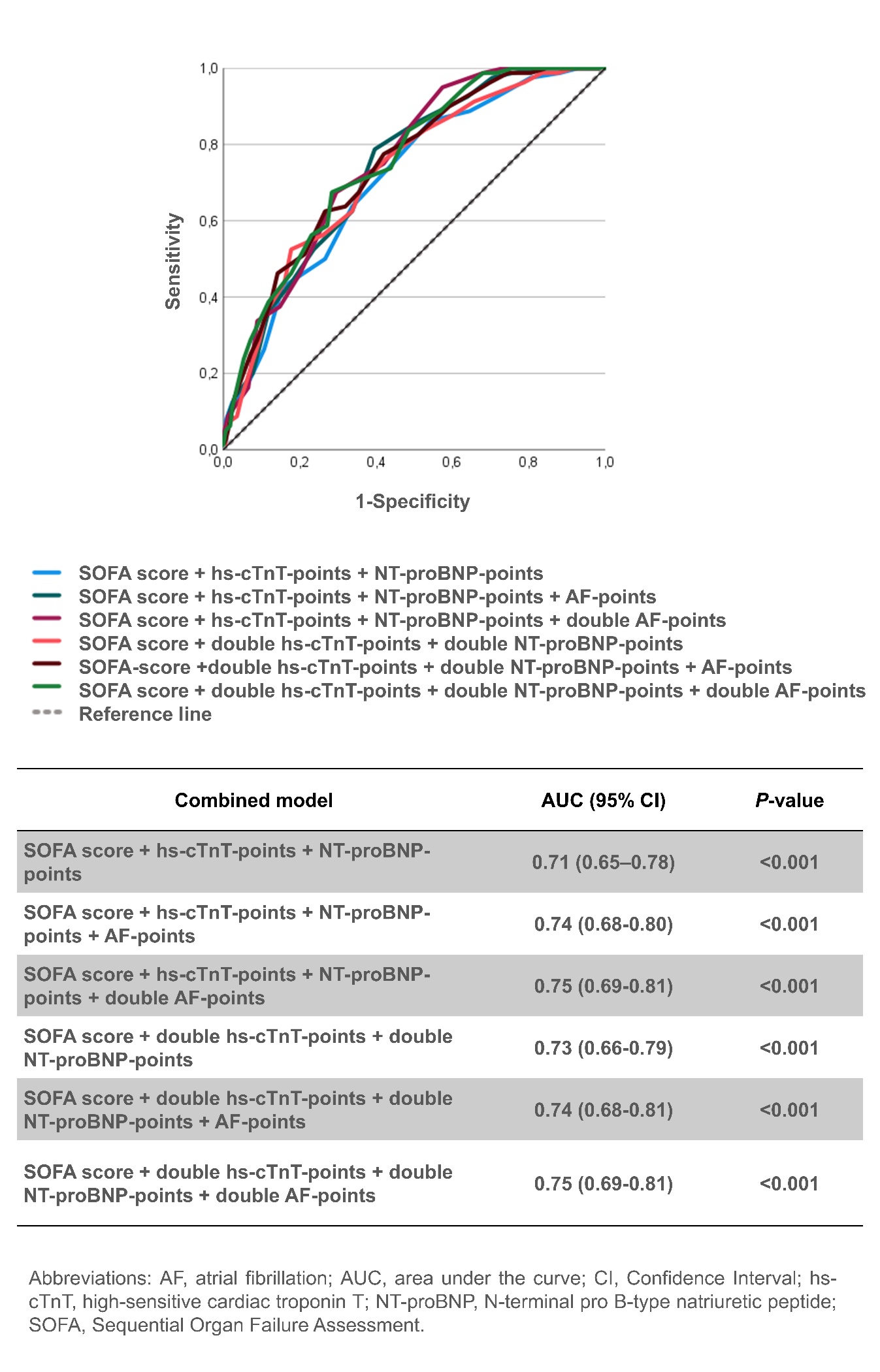


**Figure S9** Histograms showing the distribution of *(A)* the SOFA score and *(B)* the cardiac-extended SOFA (CE-SOFA) model and their associations with 30-day mortality in the training cohort. The bars are coloured to indicate patients who were alive (green) and deceased (orange) at 30 days.

**A**


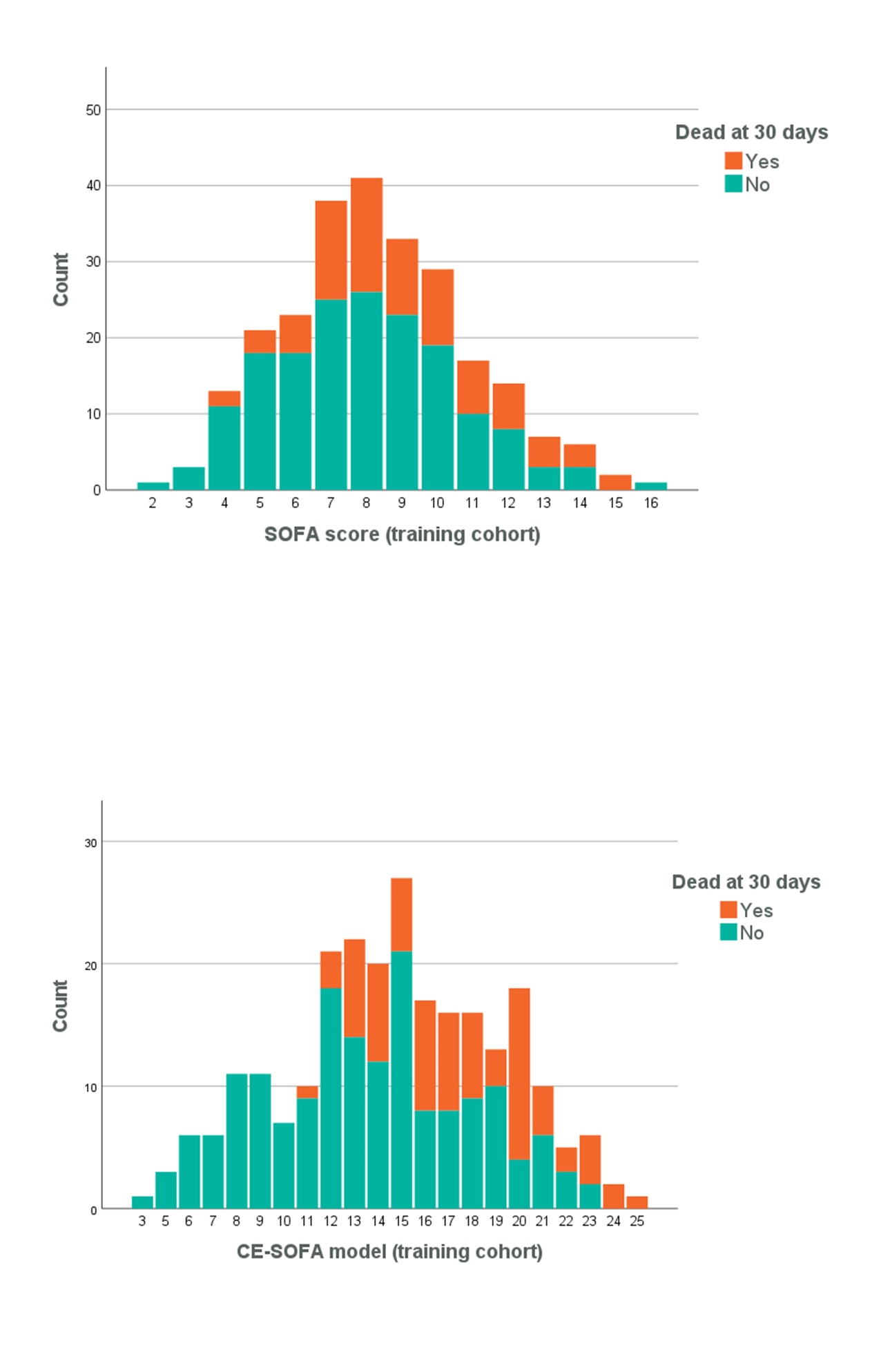


**B**

**Figure S10** Histograms showing the distribution of *(A)* the SOFA score and *(B)* the cardiac-extended SOFA (CE-SOFA) model and their associations with 30-day mortality in the test cohort. The bars are coloured to indicate patients who were alive (blue) and deceased (orange) at 30 days.


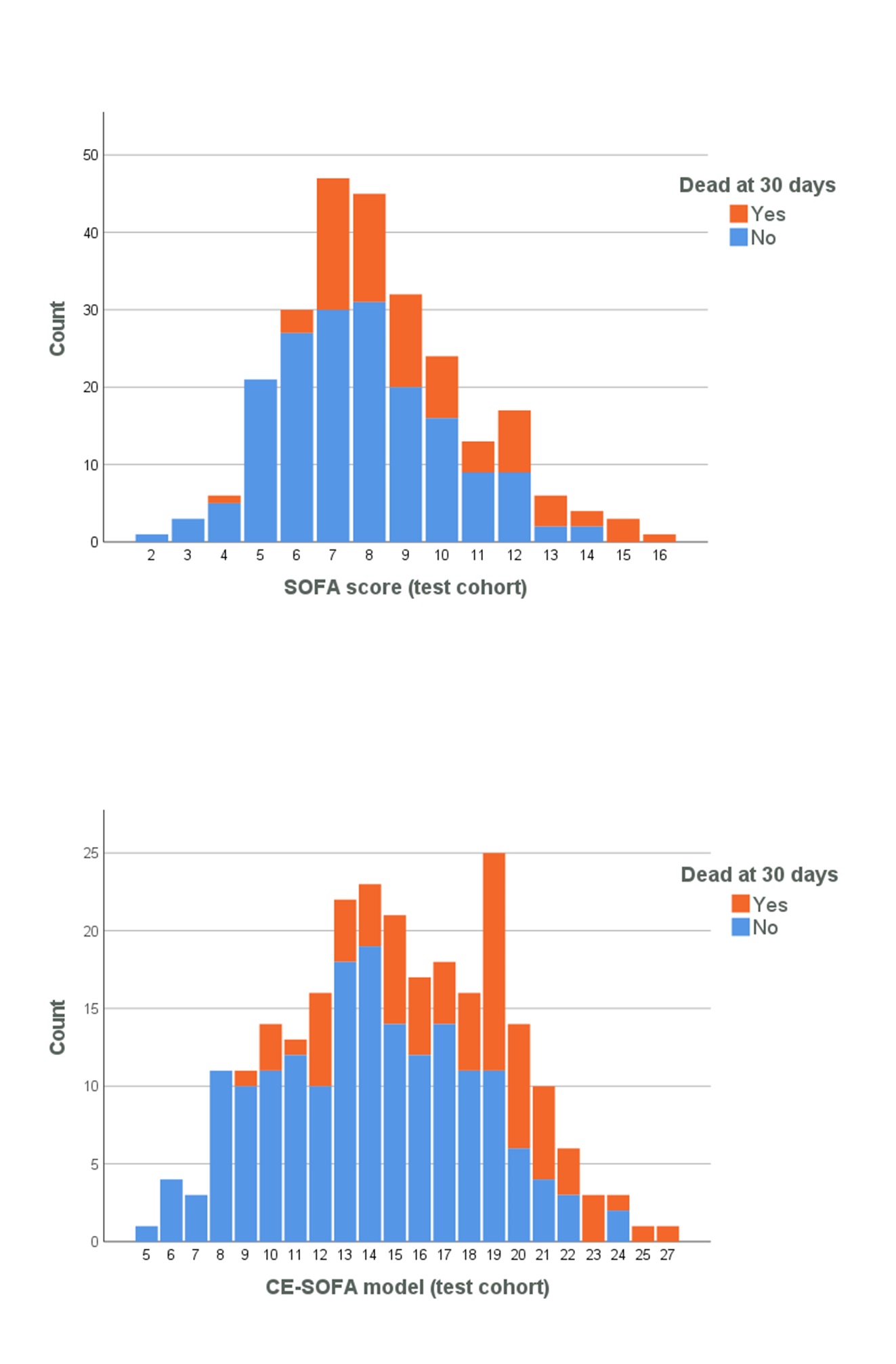


**B**

**A**

**References:**

1. Singer, M., et al., *The Third International Consensus Definitions for Sepsis and Septic Shock (Sepsis-3).* Jama, 2016. **315**(8): p. 801-10.

2. Lörstad, S., et al., *First sampled high-sensitive cardiac troponin T is associated with one-year mortality in sepsis patients and 30-365-day mortality in sepsis survivors.* Am J Med, 2023.
